# Glomerular Endothelial Cells as Instigators of Glomerular Sclerotic Diseases

**DOI:** 10.3389/fphar.2020.573557

**Published:** 2020-10-06

**Authors:** Marloes Sol, Jan A. A. M. Kamps, Jacob van den Born, Marius C. van den Heuvel, Johan van der Vlag, Guido Krenning, Jan-Luuk Hillebrands

**Affiliations:** ^1^ Department of Pathology and Medical Biology, Division of Medical Biology, University of Groningen, University Medical Center Groningen, Groningen, Netherlands; ^2^ Department of Internal Medicine, Division of Nephrology, University of Groningen, University Medical Center Groningen, Groningen, Netherlands; ^3^ Department of Pathology and Medical Biology, Division of Pathology, University of Groningen, University Medical Center Groningen, Groningen, Netherlands; ^4^ Department of Nephrology, Radboud Institute for Molecular Life Sciences, Radboud University Medical Center, Nijmegen, Netherlands

**Keywords:** Kidney glomerulus (MeSH: D007678), Glycocalyx (MeSH: D019276), Endothelial cells (MeSH: D042783), Podocytes (MeSH: D050199), Proteinuria (MeSH: D011507), Diabetic Nephropathy (MeSH: D003928), Focal Segmental Glomerulosclerosis (MeSH: D005923)

## Abstract

Glomerular endothelial cell (GEnC) dysfunction is important in the pathogenesis of glomerular sclerotic diseases, including Focal Segmental Glomerulosclerosis (FSGS) and overt diabetic nephropathy (DN). GEnCs form the first cellular barrier in direct contact with cells and factors circulating in the blood. Disturbances in these circulating factors can induce GEnC dysfunction. GEnC dysfunction occurs in early stages of FSGS and DN, and is characterized by a compromised endothelial glycocalyx, an inflammatory phenotype, mitochondrial damage and oxidative stress, aberrant cell signaling, and endothelial-to-mesenchymal transition (EndMT). GEnCs are in an interdependent relationship with podocytes and mesangial cells, which involves bidirectional cross-talk *via* intercellular signaling. Given that GEnC behavior directly influences podocyte function, it is conceivable that GEnC dysfunction may culminate in podocyte damage, proteinuria, subsequent mesangial activation, and ultimately glomerulosclerosis. Indeed, GEnC dysfunction is sufficient to cause podocyte injury, proteinuria and activation of mesangial cells. Aberrant gene expression patterns largely contribute to GEnC dysfunction and epigenetic changes seem to be involved in causing aberrant transcription. This review summarizes literature that uncovers the importance of cross-talk between GEnCs and podocytes, and GEnCs and mesangial cells in the context of the development of FSGS and DN, and the potential use of GEnCs as efficacious cellular target to pharmacologically halt development and progression of DN and FSGS.

## The Kidney and the Glomerulus

The kidneys have a vital role in fluid homeostasis and osmoregulation. Additionally, the kidneys are important for control of blood pressure and mineral metabolism. By filtering blood in the glomeruli, the kidneys produce about 150 liter glomerular filtrate per day of which 99% is reabsorbed in the tubules, to eventually generate approximately 1 liter of urine per day. By blood filtration and tubular excretion, waste products such as urea, minerals and toxic substances, are excreted from the body.

**Figure 1:**
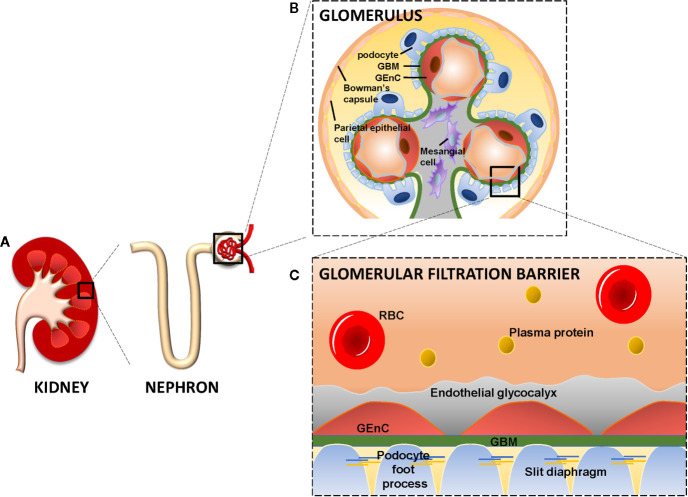
The kidney, glomerulus, and the glomerular filtration barrier. Each kidney consists of about 1 million nephrons. Each nephron consists of a glomerulus and a tubular compartment **(A)**. The glomerulus is assembled by four different cell types, namely parietal epithelial cells, glomerular endothelial cells (GEnC), podocytes (visceral epithelial cells), and mesangial cells **(B)**. GEnC and podocytes share a common extracellular matrix, the glomerular basement membrane (GBM). GEnC and their fenestrae are covered by the endothelial glycocalyx. Podocytes contain foot processes with slit diaphragms that are wrapped around the exterior of glomerular capillaries. Together, the GEnC and the endothelial glycocalyx, GBM and podocytes comprise the glomerular filtration barrier to filter the blood and remaining essential plasma proteins in the circulation **(C)**. RBC, Red Blood Cell; GBM, Glomerular Basement Membrane; GEnC, Glomerular Endothelial Cell.

The glomerulus is a network of capillary loops, known as the glomerular tuft, and is enclosed by the Bowman’s capsule. Blood flows into the glomerulus *via* the afferent arteriole and leaves the glomerulus *via* the efferent arteriole (Scott and Quaggin, 2015). The glomerulus is assembled by four different cell types: parietal epithelial cells, glomerular endothelial cells (GEnCs), podocytes (visceral epithelial cells), and mesangial cells (**Figures 1A, B**). Parietal epithelial cells line the Bowman’s capsule, where the pre-urine is collected and forwarded to the proximal tubule. GEnCs cover the luminal surface of glomerular capillaries and are the cells of the glomerulus in direct contact with the blood. GEnCs are characterized by transcellular pores (i.e., fenestrae), essential for blood filtration. At the adluminal side, GEnCs are covered with the endothelial glycocalyx, filling the fenestrae (Satchell, 2013; Scott and Quaggin, 2015; Hegermann et al., 2016) (**Figure 1C**). The endothelial glycocalyx is a gel-like layer consisting of glycoproteins, proteoglycans with bound glycosaminoglycans (GAGs) (Reitsma et al., 2007; Slater et al., 2012; Garsen et al., 2014; Dane et al., 2015) and plasma proteins loosely adherent within the meshwork of the glycocalyx. The endothelial glycocalyx prevents leakage of circulating plasma proteins by size and steric hindrance and electrostatic repulsion (Ryan and Karnovsky, 1976; Singh et al., 2007; Patrakka and Tryggvason, 2010; Friden et al., 2011; Dane et al., 2013), and inhibits adhesion and extravasation of inflammatory cells.

The endothelial glycocalyx serves as the primary sensor of wall shear stress through the initiation of signal transduction in GEnCs (Tarbell and Ebong, 2008). Wall shear stress, the hydrodynamic frictional force created from blood flow, transmits through the endothelial glycocalyx into the GEnC, leading to signal transduction that subsequently regulates the expression of Krüppel Like Factor 2 (KLF2), KLF4 and the transcription of eNOS and the production of nitric oxide (NO) which are crucial to maintain GEnC function (Dekker et al., 2006; Weinbaum et al., 2007; Ohnesorge et al., 2010; Slater et al., 2012; Dogne et al., 2018). In addition, GEnCs also function as a sink for factors essential for the regulation of the vascular tone and cross-talk with other glomerular cell types, such as vasoactive factors (endothelin-1 (ET-1), and NO) (Feliers et al., 2005; Dhaun et al., 2012).

Podocytes are specialized perivascular epithelial cells with elaborate projections called foot processes that are intimately wrapped around the exterior of glomerular capillaries (**Figures 1B, C** **)**. The foot processes leave slits between them, called slit diaphragms, which are instrumental for proper blood filtration. GEnCs and podocytes share a common extracellular matrix, referred to as the glomerular basement membrane (GBM), which separates the GEnCs from the podocytes. Together, the GEnCs and the endothelial glycocalyx, the GBM, and the podocytes constitute the glomerular filtration barrier (GFB). The GFB is responsible for size-selective and charge-dependent filtration of the blood. Small and positively charged molecules such as urea, glucose, amino acids, and minerals can pass the GFB freely, whereas circulating cells and large and negatively charged proteins, including albumin, cannot pass the GFB. Mesangial cells are located in between the capillaries and form the mesangium together with their extracellular matrix (ECM). The mesangium provides structural stability to the glomerular vasculature and modulates capillary blood flow (Scott and Quaggin, 2015). The functionality and integrity of the GFB depends on proper function of GEnCs, podocytes and mesangial cells. Dysfunction of any of the cellular or extracellular components of the GFB culminates in a decreased filtration and eventually glomerulosclerosis (Haraldsson and Nystrom, 2012; Fu et al., 2015).

## Cross-Talk Between Glomerular Cells Is Essential for Glomerular Integrity

There is a growing understanding of the interdependent relationship between GEnCs, podocytes and mesangial cells, which involves bidirectional cross-talk at a molecular level. To exemplify the importance of cross-talk between glomerular cells, the signaling of Vascular Endothelial Growth Factor A (VEGFA), Endothelin-1 (ET-1), and endothelial Nitric Oxide Synthase (eNOS) between GEnCs and podocytes are described. These molecules together form the VEGFA-eNOS/NO-ET-1 axis between GEnCs and podocytes.

### VEGFA-eNOS/NO-ET-1 Axis

**Figure 2:**
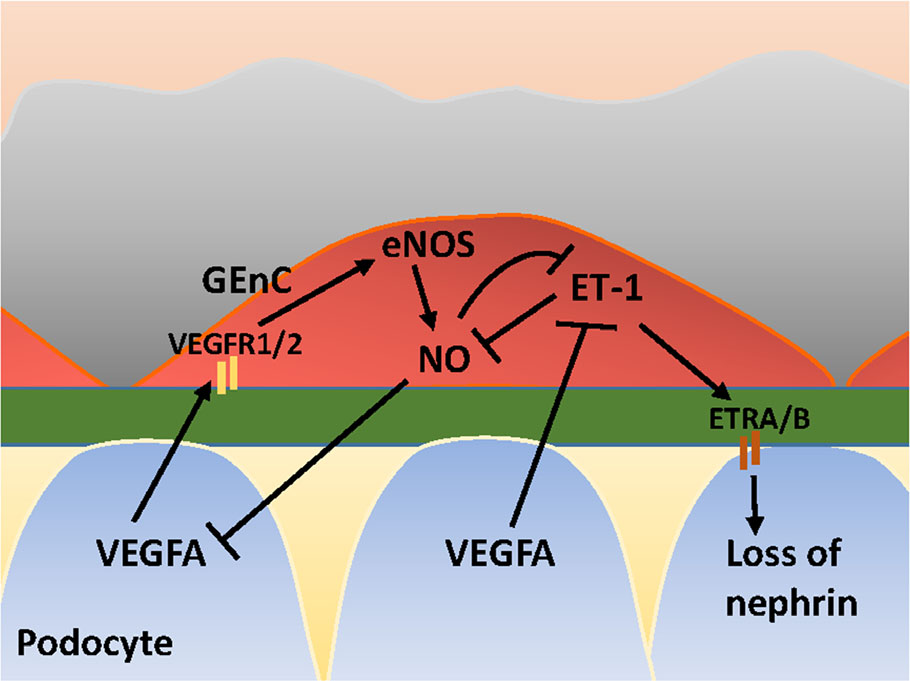
Glomerular cross-talk between GEnC and podocytes *via* the VEGFA-eNOS/NO-ET-1 axis. VEGFA, Vascular Endothelial Growth Factor A; VEGFR1/2, VEGF Receptor 1 and 2; eNOS, endothelial Nitric Oxide Synthase; NO, Nitric Oxide; ET-1, Endothelin-1; ETRA/B, ET-1 Receptor A and B. Stimulating and inhibitory effects are indicated with arrows and blunt lines, respectively.

VEGFA is synthesized by podocytes and binds to its receptors VEGFR1 and VEGFR2 expressed on GEnCs (Eremina et al., 2008). Under physiological conditions, VEGFA induces eNOS activation in GEnCs and a subsequent increase in NO production. The increase of NO may negatively regulates the amount of VEGFA produced by podocytes (Mooyaart et al., 2011). *Via* this crosstalk, the glomerular cells ensure that sufficient VEGFA is produced to maintain viability of GEnCs, without VEGFA levels rising to a level that induces sprouting angiogenesis by GEnCs. In addition to NO, VEGFA also regulates ET-1 production by GEnCs, since VEGFA blockage in podocytes induces ET-1 release from GEnCs (Collino et al., 2008). GEnCs are considered the principal source of ET-1 within the glomeruli (Herman et al., 1998). ET-1 exerts its effect *via* ET-1 receptors (ETR) A and ETRB. Low levels of ET-1 induce an increase in NO, whereas high levels of ET-1 inhibit NO production (Watschinger et al., 1995; Dong et al., 2005; Sud and Black, 2009). ET-1 release from GEnCs associates with cytoskeleton redistribution with a decrease of nephrin in podocytes (Lenoir et al., 2014; Yuan et al., 2019). NO, in its turn, inhibits ET-1 expression (Khimji and Rockey, 2010) and exerts protective effects in podocytes (Sun et al., 2013). An illustration of cross-talk between GEnCs and podocytes in the VEGFA-eNOS/NO-ET-1 axis is provided in **Figure 2**. Next to the effect of ET-1 on podocytes, ET-1 also exerts effects on mesangial cells. ETRA signaling is associated with inflammation, contraction and proliferation of mesangial cells (Barton and Sorokin, 2015), and fibrosis. ETRB signaling has a reciprocal effect and is associated with vasorelaxation *via* eNOS-derived NO release (Barton and Yanagisawa, 2008).

## Glomerular Sclerotic Diseases: Histopathology of FSGS and DN

DN is a long-term complication of both type 1 and type 2 diabetes mellitus and develops in 20%–40% of all diabetes mellitus patients (Rossing et al., 2018). DN, together with Focal Segmental Glomerulosclerosis (FSGS), is the most important cause of chronic kidney disease (CKD). Two types of FSGS exist: primary (or idiopathic) FSGS and secondary FSGS. In primary FSGS, which comprises 80% of all FSGS cases, the etiology is unknown. Secondary FSGS is induced by a preexisting pathologic condition, e.g., hypertension (Jefferson and Shankland, 2014), a viral infection, such as human immunodeficiency virus, drug-induced, or induced by genetic mutations (Lim et al., 2016). In case of primary or mutation-induced FSGS, mutations in genes encoding proteins expressed in podocytes, which are mostly related to slit diaphragm structure, the actin cytoskeleton, or foot processes, such as nephrin (NPHS1), podocin (NPHS2), actinin α4 (ACTN4), and TRPC6 are commonly observed (Lim et al., 2016). No mutations are known in GEnC-specific genes that would cause FSGS.

**Figure 3:**
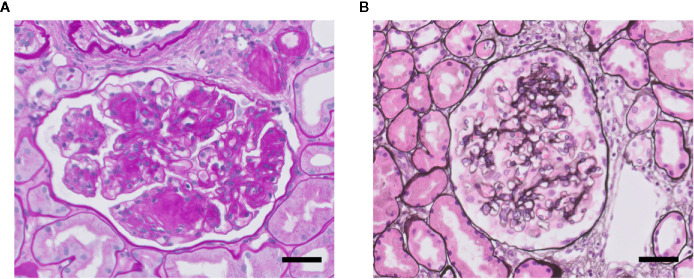
Glomerulosclerosis in DN (A) and FSGS (B). Light microscopy photomicrographs of a glomerulus showing DN with characteristic nodular mesangial expansion (Kimmelstiel-Wilson lesions) (Periodic acid–Schiff staining) **(A)**, and of a glomerulus with mild FSGS (methenamine-silver staining) **(B)**. Nuclei are stained in blue. Both glomeruli presenting glomerulosclerosis with increased glomerular extracellular matrix deposition and obliteration of capillaries. Scale bars represent 50 µm.

FSGS and overt diabetic nephropathy (DN) both are characterized by scarring (sclerosis) of the glomerular tuft, i.e., glomerulosclerosis (**Figure 3**). Glomerulosclerosis causes obliteration of the glomerular capillaries eventually (Fioretto and Mauer, 2007; De Vriese et al., 2018). In FSGS, only a fraction of the glomeruli (i.e., focal) is affected in a segmental manner, i.e., part of a glomerulus is affected. Sclerosis in FSGS is characterized by deposition of extracellular matrix (ECM) at the capillary loops. DN is the specific histopathology associated with reduced renal function in patients suffering from diabetic kidney disease (Yamanouchi et al., 2020). Overt DN comprises diffuse and sometimes nodular glomerulosclerosis in many glomeruli, caused by mesangial cell proliferation and mesangial sclerosis, and develops primarily in patients with proteinuria. Of note, nonproteinuric diabetic kidney disease also exists and which is characterized by minor histopathological changes without DN and with better prognosis compared with proteinuric diabetic kidney disease (Yamanouchi et al., 2020). So, particularly FSGS but also DN are accompanied by proteinuria (macroalbuminuria: >300 mg/gr creatinine), as well as by glomerular hypertension and hyperfiltration, and activation of glomerular inflammatory pathways (Fioretto and Mauer, 2007; Reidy and Kaskel, 2007). At the ultrastructural level, damage to podocytes and GEnCs is observed. Podocyte injury is observed as extensive effacement of the foot processes, ultimately leading to detachment of podocytes from the GBM (podocyte loss). GEnC dysfunction is characterized morphologically as a reduction of the endothelial glycocalyx, loss of fenestrae, widening of the subendothelial space, and swelling of the cytoplasm (Weil et al., 2012; Eleftheriadis et al., 2013; Morita et al., 2015; Taneda et al., 2015; Boels et al., 2016). In many patients, DN and FSGS progresses into end-stage renal disease (ESRD). Therapy resistance and the failure to adequately treat proteinuria, a glomerular inflammatory phenotype and hypertension are the main reasons for progression towards ESRD (Kiffel et al., 2011; Collins et al., 2013). Renal replacement therapy (dialysis or kidney transplantation), is the only effective treatment to postpone premature death in ESRD patients (Collins et al., 2014).

## GEnC Dysfunction in DN and FSGS

GEnC dysfunction is important in the pathogenesis of glomerular sclerotic diseases, including FSGS and overt DN. GEnCs, covered by a thick glycocalyx, form the first cellular barrier in direct contact with all circulating factors. Changes in these circulating factors, such as high glucose levels and advanced glycation end-products, can induce GEnC dysfunction (Singh et al., 2011; Singh et al., 2013; Peng et al., 2016). In FSGS, the development of mesangial matrix expansion and sclerosis by parietal epithelial cells appears to be secondary to podocyte injury, whereas in DN mesangial matrix expansion is the key morphologic finding (Najafian et al., 2015). It is likely that GEnC dysfunction precedes and possibly also contributes to podocyte damage and mesangial expansion. In the past decade, evidence has been provided that also GEnC dysfunction is present and plays an important role in FSGS and DN development. GEnC dysfunction occurs in the early stages of FSGS and DN, and is sufficient to cause podocyte injury, proteinuria and activation of mesangial cells, as will be discussed in detail below. An interdependent relationship between GEnCs, podocytes and mesangial cells exists, which involves bidirectional cross-talk with intercellular signaling. Disturbed molecular cross-talk involving for example endothelial nitric oxide synthase (eNOS) may result in reduced GEnC-derived NO exposure to podocytes and can induce podocyte damage, and eventually compromise glomerular integrity (Yuen et al., 2012). Therapies aiming to prevent endothelial injury have shown to reduce DN in animal models. For example ETRA blockers have shown to restore the endothelial glycocalyx and to reduce albuminuria in diabetic mice (Boels et al., 2016). In diabetic patients, the ETRA blocker atrasentan reduced urinary albumin to creatinine ratios (Lin et al., 2018). Furthermore, renal elevation of cGMP, a key messenger for NO signaling, resulted in a reduction of glomerulosclerosis in rats with DN (Boustany-Kari et al., 2016). Given that GEnCs are the first cells exposed to changes in circulating factors and that GEnC behavior directly influences podocyte function, it is conceivable that GEnC dysfunction may culminate in podocyte damage and mesangial activation. It is, however, elusive which molecular mechanisms underlie GEnC dysfunction and the subsequent altered cross-talk with podocytes and mesangial cells. To develop new treatment options in order to halt the progression of glomerular sclerotic disease, a deeper understanding of the pathogenetic mechanisms underlying GEnC dysfunction and the disturbed cross-talk is required. Hereunder, it is described that GEnC dysfunction comprises multiple facets and is a pivotal and early factor in the development of glomerulosclerosis and is at the basis of developing proteinuria, podocyte dysfunction and mesangial expansion in FSGS and DN.

### Compromised Endothelial Glycocalyx in DN and FSGS

Healthy GEnCs are covered with an endothelial glycocalyx. The endothelial glycocalyx consists of glycoproteins, glycolipids and proteoglycans with bound GAGs. Proteoglycans with bound GAGs, of which heparan sulphate and hyaluronan constitute up to 90%, are the main contributors to the function and structure of the endothelial glycocalyx (Reitsma et al., 2007; Slater et al., 2012; Garsen et al., 2014; Dane et al., 2015). In DN and FSGS, the endothelial glycocalyx is reduced, characterized by a loss of essential GAGs, including heparan sulphate and hyaluronan, and reduced thickness (Nieuwdorp et al., 2006a; Kuwabara et al., 2010; Satoh et al., 2010; van den Berg et al., 2019). Environmental factors, such as elevated levels of glucose, oxidative stress, or inflammatory stimuli, can modulate the endothelial glycocalyx (Singh et al., 2011; Singh et al., 2013; Kolarova et al., 2014). Inflammatory mediators like cytokines and chemokines cause degradation of the endothelial glycocalyx. Under physiological conditions, adhesion molecules on endothelial are covered by the endothelial glycocalyx, and only become accessible to leukocytes upon degradation of the glycocalyx (Kolarova et al., 2014). *In vivo*, intravenous administration of the bacterial heparan sulphate-degrading enzyme heparinase enhances leukocyte adherence to endothelial cells (Constantinescu et al., 2003). High glucose and oxidative stress cause a reduction of heparan sulphate in the endothelial glycocalyx on GEnCs *in vitro* (Singh et al., 2011; Singh et al., 2013). Furthermore, high glucose reduces GAG biosynthesis in GEnCs (Singh et al., 2011). Reduction of heparan sulphate culminates in increased passage of albumin across a GEnC monolayer (Singh et al., 2011; Singh et al., 2013). In line with these *in vitro* data, a reduced endothelial glycocalyx instantly causes proteinuria *in vivo* (Gil et al., 2012). Preservation of the endothelial glycocalyx by the genetic deletion of the heparan sulphate-degrading enzyme heparanase prevents proteinuria and kidney failure in experimental DN and glomerulonephritis (Gil et al., 2012; Garsen et al., 2016a). Loss of endothelial hyaluronan and thereby the endothelial glycocalyx induced by an endothelial-specific deletion of the hyaluronan synthesis enzyme hyaluronan synthase 2 (HAS2) (van den Berg et al., 2019) or by treatment with the hyaluronan-degrading enzyme hyaluronidase (Meuwese et al., 2010) also induces proteinuria (Meuwese et al., 2010; van den Berg et al., 2019) and progressive glomerulopathy (van den Berg et al., 2019), phenocopying the events in DN. In addition to the induction of leukocyte adherence and proteinuria, degradation of the endothelial glycocalyx also compromises GEnC signaling *via* the loss of mechanosensing. Fluid shear stress induces the production of NO in endothelial cells *via* activation of eNOS (Boo et al., 2002). Fluid shear stress-induced NO production is almost completely inhibited upon enzymatic removal of heparan sulphate in the endothelial glycocalyx (Florian et al., 2003), due to loss of eNOS activation. Impaired eNOS activation has negative effects on both GEnCs and podocytes *in vivo* as this results in GEnC dysfunction and disturbed cross-talk with podocytes (Yuen et al., 2012). A reduced endothelial glycocalyx on GEnCs, in response to noxious stimuli, clearly induces glomerular inflammation, proteinuria, and disturbs GEnC signaling. Loss of the endothelial glycocalyx coincides with coagulation activation (Nieuwdorp et al., 2006b) and could possibly also be linked with complement activation (Boels et al., 2013), which is described elsewhere (Nieuwdorp et al., 2006b; Boels et al., 2013) and will not further be addressed here.

### Compromised Barrier Function by Endothelial Cell-Selective Adhesion Molecule (ESAM)

The barrier function of GEnCs in FSGS or DN is mainly compromised by a reduction of the endothelial glycocalyx but additional factors that contribute to an increased permeability have been described as well. The altered expression of endothelial cell-selective adhesion molecule (ESAM) has been implied in the loss of the endothelial cell barrier in DN. ESAM is a surface protein laterally expressed on GEnCs that is part of the endothelial tight junctions, and mediates the interaction between endothelial cells. ESAM expression is reduced in the early course of DN (4 weeks) and is associated with increased vascular permeability *in vitro*. *In vivo*, genetic ablation of ESAM causes proteinuria, a decrease in GEnC fenestrations and an increased space between GEnCs through expanded tight junctions, while no structural changes are observed in podocytes, the GBM and mesangium (Hara et al., 2009). Therefore, these observations provide evidence that solely GEnC dysfunction (induced by ESAM deficiency) already leads to glomerular paracellular albumin leakage with preserved podocyte structure (Hara et al., 2009).

### Pro-Inflammatory Phenotype

GEnC dysfunction also contributes to glomerulosclerosis *via* obtaining a pro-inflammatory phenotype without having direct effects on podocytes and mesangial cells. Inflammatory pathways are involved in the pathogenesis of DN and FSGS (Navarro-Gonzalez et al., 2011; Wada and Makino, 2013; Moreno et al., 2018; Wilkening et al., 2020). Inflammation-related molecules and pathways (but without pronounced inflammation) may promote fibrotic and proliferative responses of mesangial cells, culminating in glomerulosclerosis (Furuta et al., 1993; Fogo, 2007). GEnC activation plays an important role in glomerular leukocyte infiltration as GEnC activation enables leukocyte rolling, adhesion, arrest and transmigration across the endothelial cell lining (Ley et al., 2007). Upon GEnC activation, the expression of chemokines and adhesion molecules on the cell surface of GEnCs, such as E-selectin (Hirata et al., 1998), intercellular adhesion molecule 1 (ICAM-1) and monocyte chemoattractant protein 1 (MCP-1), are increased (Rao et al., 2017). The high glucose-induced toxic metabolites advanced glycation end-products (AGEs), induce the expression of ICAM-1 and MCP-1 in a Rho-kinase dependent manner. AGE-induced activation of Rho-kinase could be a result of activation of the receptor for AGEs (RAGE) (Hirose et al., 2010). Also ET-1 can activate Rho-kinase in endothelial cells (Gien et al., 2013). Blockage of Rho-kinase in DN mice reduces the expression of ICAM-1 and MCP-1, and ablates concomitant glomerular infiltration of macrophages and glomerulosclerosis. Since macrophages also display Rho-kinase, an endothelial-specific inducible Rho-kinase gene targeting approach would be needed to confirm the role of endothelial Rho-kinase in the increased expression of ICAM-1 and MCP-1 in DN. This implies that AGEs-induced expression of adhesion molecules on GEnCs plays a key role in the development of diabetic glomerulosclerosis (Rao et al., 2017). Indeed, inhibition of AGEs reduces glomerulosclerosis in diabetic mice (Wilkinson-Berka et al., 2002; Forbes et al., 2003). In addition to the increased expression of adhesion molecules, GEnCs show a reduced expression of endothelial-specific molecule-1 (ESM-1), already in very early stages of DN. Under physiological conditions, GEnCs constitutively express ESM-1 that functions as an anti-inflammatory molecule and inhibits migration and rolling of leukocytes. Four weeks after the induction of diabetes, before the development of histological glomerular changes indicative of DN, ESM-1 expression was decreased in glomeruli of DN-susceptible mice compared to glomeruli of DN-resistant mice. These observations demonstrate that in early stages of DN, GEnCs display a pro-inflammatory phenotype which precedes glomerular damage (Zheng et al., 2017).

### Mitochondrial Damage

In DN and FSGS, GEnCs display oxidative mitochondrial DNA lesions and mitochondrial oxidative stress, which is associated with loss of GEnC fenestrations (Daehn et al., 2014; Qi et al., 2017) and a loss of the endothelial glycocalyx (Ebefors et al., 2019). Mitochondrial oxidative stress in GEnCs was mediated by release of ET-1 by podocytes and the subsequent paracrine ETRA activation in GEnCs (Daehn et al., 2014; Ebefors et al., 2019). ET-1 induced an increase in heparanase mRNA expression in GEnCs *in vitro*, which could explain the loss of the endothelial glycocalyx upon release of ET-1 by podocytes *in vivo* (Ebefors et al., 2019). Mitochondrial oxidative stress was only observed in GEnCs and not in podocytes in streptozotocin (STZ)-induced DN (Qi et al., 2017). Interestingly, mitochondrial damage in GEnCs preceded podocyte loss, proteinuria, and glomerulosclerosis in adriamycin-induced FSGS and STZ-induced DN (Daehn et al., 2014; Qi et al., 2017). Scavenging of mitochondrial superoxide by systemic administration of the mitochondria-targeted potent antioxidant mitoTEMPO prevented GEnC mitochondrial oxidative stress (Daehn et al., 2014; Qi et al., 2017), the loss of fenestrations (Qi et al., 2017) and the loss of the endothelial glycocalyx (Ebefors et al., 2019). Attenuation of GEnC mitochondrial stress results in ameliorated podocyte loss, demonstrating that mitochondrial damage in GEnCs and the resulting production of mitochondrial superoxide are important triggers for podocyte loss (Daehn et al., 2014; Nagasu et al., 2016; Qi et al., 2017).

### eNOS Inactivation

eNOS inactivation, due to impaired dimerization and phosphorylation, has been suggested to play an important role in experimental DN (Cheng et al., 2012). In mice, resistant for adriamycin-induced glomerulopathy, administration of adriamycin induced massive proteinuria and severe glomerulosclerosis upon eNOS deficiency. This observation shows that loss of eNOS increases the susceptibility for the development of adriamycin-induced nephropathy. GEnC dysfunction, observed as loss of CD31 and apoptosis, appeared 3 days after adriamycin administration. Notably, podocyte damage (i.e., loss of synaptopodin expression and apoptosis), occurred only after 7 days, demonstrating that GEnC dysfunction preceded podocyte damage in this model (Sun et al., 2013). Part of these *in vivo* results could be explained by adriamycin’s ability to induce inflammatory effects (Abou El Hassan et al., 2003). In line with these findings it has been shown that eNOS prevents heparanase expression and the development of proteinuria in adriamycin-induced experimental FSGS (Garsen et al., 2016b). *In vitro*, conditioned medium from eNOS-overexpressing microvascular endothelial cells protected podocytes from TNF-α-induced synaptopodin loss, suggesting that “healthy” GEnCs protect podocytes from an inflammatory insult in a paracrine manner by secreting protective mediators. Which mediators are secreted by GEnCs and how these mediators affect podocytes is not known (Sun et al., 2013).

### Disturbed Crosstalk in the VEGFA-eNOS/NO-ET-1

Disturbances in paracrine signaling of VEGFA, eNOS/NO, and ET-1 between podocytes to GEnCs are critical and may compromise glomerular integrity. Either increased or decreased VEGFA expression, decreased eNOS signaling and increased ET-1 signaling are all implicated in glomerular pathology. In mice, gain of VEGFA in podocytes and lack of eNOS causes the development of proteinuria and nodular glomerulosclerosis (Veron et al., 2014). Podocyte-specific deletion of VEGFA causes GEnC damage, observed as swelling of GEnCs, necrosis and culminating in capillary obliteration (Eremina et al., 2008) and loss of fenestrae (Eremina et al., 2003). Additionally, podocyte-specific deletion of VEGFA also causes a loss of GEnCs in diabetic mice (Sivaskandarajah et al., 2012). Whole-body deletion of VEGFR2 results in a loss of viable GEnCs (Sison et al., 2010). Also podocyte-specific VEGFA overexpression results in loss of GEnCs and collapse of capillary loops (Eremina et al., 2003) and causes advanced DN with endothelial swelling (Veron et al., 2011), suggesting the existence of a delicate balance between the protective and deleterious effects of VEGFA, depending on the strength of signaling. Deletion of eNOS causes GEnC dysfunction and subsequently podocyte damage (Yuen et al., 2012). Administration of NO to cultured podocytes increases the production of cyclic guanosine monophosphate (cGMP), which controls the cytoskeletal structure of podocytes and limits podocyte retraction (Sharma et al., 1992). Deletion of eNOS and decreased availability of NO probably causes decreased cGMP production and subsequent podocyte retraction and foot process effacement. Maintenance of endothelial eNOS levels by the essential eNOS cofactor tetrahydrobiopterin ameliorates DN (Kidokoro et al., 2013). Furthermore, treatment with sepiapterin, a stable precursor of the eNOS cofactor tetrahydrobiopterin or L-arginine, the nitric oxide precursor induces a correction of eNOS dimerization and phosphorylation and decreases albuminuria (Cheng et al., 2012). In a recent paper, it was shown that ET-1 induces heparanase expression in podocytes, which was associated with a reduced glomerular endothelial glycocalyx in experimental diabetes and which could be prevented in a podocyte-specific ETR deficient mouse model nephropathy (Garsen et al., 2016c). The mechanisms underlying the trafficking of podocyte-derived VEGFA and heparanase against the filtration direction remain to be identified, but may involve heparan sulfate present in the GBM.

These studies demonstrate that the VEGFA-eNOS/NO-ET-1 signaling pathway is important for intraglomerular cross-talk between podocytes and GEnCs, and the strength and direction of signaling is critical for glomerular health. Disturbed cross-talk causes glomerular damage. GEnCs are the first cells in contact with all circulating factors in the blood. It is therefore likely that GEnC dysfunction, culminating in altered secretion of signaling molecules, occurs prior to, and is in fact (partly) responsible for podocyte damage and activation of mesangial cells. GEnC dysfunction might therefore be a leading initiating factor in the development of both FSGS and DN.

### Other Aberrant Molecular Signaling and Expression Patterns

#### LRG1 and Enhancement of TGF-β/ALK1 Signaling

Recently, transcriptome profiling of GEnCs obtained from diabetic mice showed increased gene expression of leucine-rich α-2-glycoprotein (LRG1) in early stages of DN (Fu et al., 2018). LRG1 is a protein present in the glomeruli and is predominantly expressed by GEnCs. LRG1 is involved in angiogenesis and the pathogenesis of DN by enhancement of endothelial Tumor Growth Factor β (TGF-β)/activin receptor-like kinase 1 (ALK1) signaling. TGF-β signaling has previously been found to be involved in the pathogenesis of DN by promoting cell hypertrophy, ECM accumulation in the mesangium, and increasing glomerular permeability (Chang et al., 2016). Global genetic ablation of LRG1 led to a reduction of oxidative damage and glomerular angiogenesis in diabetic mice. Concomitantly, podocyte foot process effacement, podocyte loss, proteinuria, and glomerulosclerosis were attenuated. These results exemplify that alterations in GEnC gene expression and molecular pathways in early disease mediate podocyte damage and glomerulopathy (Hong et al., 2019). How increased LRG1 expression and TGF-β signaling in GEnCs specifically relate to podocyte damage was not addressed in these studies.

#### GEnC-Derived Exosomes

As a consequence of high glucose concentration, GEnCs show an increased secretion of exosomes containing TGF-β1 mRNA. *In vitro*, these exosomes induced mesangial cells to proliferate and produce ECM (Wu et al., 2016) and caused the induction of epithelial-mesenchymal-transition in podocytes (Wu et al., 2017). Injection of exosomes, derived from high glucose-treated GEnCs *in vitro*, caused glomerulosclerosis in mice (Wu et al., 2016). These studies together suggest that high glucose-induced GEnC dysfunction increases the production of GEnC exosomes, which induce phenotypic changes in mesangial cells and podocytes *in vitro*, and culminate in glomerulosclerosis *in vivo* (Wu et al., 2016).

#### Hypoxia-Induced Dysregulation of GEnCs

DN is associated with renal cortical hypoxia (O’Neill et al., 2015). Hypoxia and concomitant dysregulation of hypoxia-regulated transcriptional mechanisms in GEnCs are associated with the pathogenetic mechanisms involved in both FSGS and DN development. Endothelial PAS domain-containing protein 1 (EPAS1) is an isoform of hypoxia inducible factor (HIF), also known as HIF-2α. Endothelial-specific deletion of EPAS1 induced the loss of GEnC fenestrations and enhanced endothelial swelling in experimental hypertension-induced secondary FSGS. Additionally, GEnC dysfunction was associated with podocyte foot process effacement and worsening of proteinuria and glomerulosclerosis. In the presence of hypertension and EPAS1, podocyte lesions were not observed, demonstrating that aberrant EPAS1-mediated endothelial signaling associates with podocyte damage and exacerbates FSGS (Luque et al., 2017). Potential mechanisms for aforementioned results include a direct effect of EPAS1 on endothelial-dependent vasoreactivity and modulation of glomerular pressure resulting in hyperfiltration, as mechanical stress is thought to contribute to FSGS. Hyperfiltration results in glomerular hypertrophy, culminating in loss of podocytes and aggravation of mechanical stress and glomerular damage. Furthermore, EPAS1 was previously shown to associate with the assembly of intercellular adherens junctions and enhanced endothelial barrier integrity (Gong et al., 2015). The involvement of dysregulation of hypoxia-associated mechanisms in GEnCs in the pathogenetic pathways leading to glomerular disease is further substantiated by a study showing that endothelial-specific knockout of hypoxia inducible factor 1α (HIF1α) prevents the development of proteinuria and collagen deposition in hypertensive FSGS (Luo et al., 2015). These and the previous mentioned results show that HIF1α is detrimental, whereas EPAS1/HIF2α confers protection in glomerular disease. An explanation could be that the target genes of HIF1α and HIF2α differ in a context-dependent manner (Dengler et al., 2014). Collectively, the aforementioned studies show that disturbed hypoxia-driven signaling in GEnCs contributes to the pathogenesis of glomerular damage in FSGS and DN.

### GEnC Plasticity: Endothelial-to-Mesenchymal Transition

GEnC dysfunction can induce the process of endothelial-to-mesenchymal transition (EndMT). Whether EndMT is an initiating event in glomerulosclerosis, and to which extent EndMT contributes to glomerulosclerosis is not known. EndMT is a process in which endothelial cells show an abrogated endothelial phenotype (such as loss of the expression of endothelial cell markers CD31 and VE-cadherin) and loss of endothelial characteristics such as an increased vascular permeability. Loss of endothelial marker expression coincides with an increase of mesenchymal marker expression such as α-smooth muscle actin (αSMA) and fibroblast specific protein 1 (FSP-1), and the production of ECM proteins (Dejana et al., 2017). In general, endothelial cells are suggested to contribute to the number of activated fibroblasts *via* EndMT. EndMT most probably contributes to fibrosis and is observed in cardiac and cancer-related fibrosis (Zeisberg et al., 2007), fibro-proliferative vascular disease (Moonen et al., 2015), but also in experimental kidney disease as shown in streptozotocin (STZ)-induced DN, unilateral ureteral obstruction, and a mouse model for Alport’s syndrome (Zeisberg et al., 2008). In these models, ~30%–50% of the activated fibroblasts co-express the endothelial cell marker CD31 and mesenchymal markers, such as αSMA and FSP-1 (Zeisberg et al., 2008). In lineage tracing experiments in STZ-induced diabetic mice, interstitial endothelial cells acquired a more mesenchymal-like phenotype by expressing αSMA, already early in development of renal interstitial fibrosis (Li et al., 2009). Also in glomeruli of DN patients, EndMT is observed as demonstrated by co-expression of endothelial and mesenchymal markers (Peng et al., 2016; Liu et al., 2018). High glucose conditions and advanced oxidation protein products will stimulate GEnCs to undergo EndMT (Liang et al., 2016; Peng et al., 2016; Shang et al., 2017). Together, aforementioned observations provide evidence that GEnCs can acquire a mesenchymal-like phenotype and may contribute to glomerular fibrosis in DN. The process of EndMT is shown to be controlled by autophagy in endothelial cells (Patschan et al., 2016; Wang et al., 2017). In diabetic mice, deletion of autophagy in endothelial cells induced by the endothelial-specific genetic deletion of Autophagy-Related Gene 5 (ATG5) caused endothelial cell lesions, podocyte foot process broadening and effacement, and an increase of microalbuminuria. These results exemplify the tight intercellular cross-talk between GEnC and podocytes, in which GEnC dysfunction (induced by ATG5 deficiency) leads to podocyte injury (Lenoir et al., 2015).

## Epigenetic Modifications: A Potential Mechanism Involved in GEnC Dysfunction

The above mentioned facets of GEnC dysfunction in FSGS and DN associate with altered gene and protein expression. A quiescent endothelial phenotype is harbored by tight regulation of the endothelial transcriptome, i.e., the full array of mRNA transcripts produced (Brooks et al., 2002; Passerini et al., 2004; Gimbrone and Garcia-Cardena, 2013). Epigenetic mechanisms are involved in this regulation of the transcriptome of cells (Eccleston et al., 2013). Epigenetic modifications can cause changes in gene expression, without changing the DNA sequence (Gibney and Nolan, 2010) and are self-perpetuating, dynamic, and reversible in response to the environment (Beckerman et al., 2014). Many factors can influence epigenetic profiles, including hyperglycemia, hypoxia, and inflammation (Lu et al., 2017). Epigenetic modifications can either be beneficial, or hamper GEnC function by changing the transcriptome, resulting in GEnC dysfunction and potentially disturbed cross-talk and pathogenesis of FSGS and DN.

Epigenetic modifications include DNA methylation and histone modifications. In general, DNA methylation is associated with gene repression by changing the biophysical characteristics of the DNA to bind transcription factors. DNA methylation can also inhibit gene expression *via* methyl binding proteins, which in turn recruit transcriptional co-repressors. DNA methylation at genes can modulate transcriptional elongation and alternative splicing (Gibney and Nolan, 2010; Lu et al., 2017).

In addition to DNA methylation, epigenetic mechanisms also include modifications of histones. The best-characterized histone modifications involve methylation, acetylation, and phosphorylation. Histone modifications stably alter the conformation of chromatin, and thereby either enhance or inhibit gene transcriptional activity depending on the type of modification and the position of the modified residue within the histone (Kouzarides, 2007; Berger et al., 2009). DN is associated with aberrant DNA methylation in proximal tubules and peripheral blood cells (Maghbooli et al., 2014), and DNA methylation is recently shown to be present in GEnCs (Fu et al., 2018). Histone modifications have previously been shown to be involved in the pathogenesis of DN and FSGS (Sun et al., 2017; Majumder et al., 2018), but not much is known about altered histone modification patterns in GEnCs in DN or FSGS. Recently, transcriptome profiling of GEnCs obtained from diabetic mice with early DN, showed that many of the genes with decreased expression were involved in epigenetic regulation, suggesting altered epigenetic regulation in GEnCs in early stages of DN (Fu et al., 2018). Lysine-specific demethylase 6A (KDM6a), also known as Ubiquitously Transcribed Tetratricopeptide Repeat X Chromosome (UTX) was one of the genes found to be downregulated. KDM6a is a histone demethylase that specifically demethylates lysine 27 of histone 3. Methylation of lysine 27 of histone 3 (H3K27me3), mediated by the methyltransferase Enhancer of Zeste Homolog 2 (EZH2), is associated with gene repression (Tan et al., 2014). The role of EZH2 and H3K27me3 in GEnCs in DN and FSGS is yet unknown. In podocytes, H3K27me3 was previously shown to be decreased in DN, which associated with the extent of podocyte damage due to activation of Notch signaling and loss of quiescence (Majumder et al., 2018). Previous studies showed that EZH2 plays a role in endothelial homeostasis and is a modulator of a number of endothelial cell functions, such as endothelial-leukocytes interactions and angiogenesis (Dreger et al., 2012; Maleszewska et al., 2016). This is indicative for a role of altered epigenetic modifications in GEnCs resulting in aberrant and pathologic gene expression contributing to the pathogenesis of DN. Alteration of epigenetic modifications is shown to be beneficial. For example, inhibition of the demethylases Jumonji C domain–containing demethylases (JMJD3) and UTX attenuated podocyte injury in diabetic mice (Majumder et al., 2018). Also in an unilateral ureteric obstruction mouse model, inhibition of EZH2 and H3K27me3 attenuated renal fibrosis (Zhou et al., 2016). Our current knowledge about the contribution of an altered epigenetic landscape to GEnC dysfunction and disturbed cross-talk in DN and FSGS is limited. Therefore, expanding our knowledge on the potential causative role of epigenetic modifications in GEnCs is highly needed. Herewith, specific mediators involved in epigenetic pathways involved in GEnC dysfunction and disturbed cross-talk can be considered potential targets for future therapies in the pathogenesis of DN and FSGS.

## Summary and Future Perspectives

**Figure 4:**
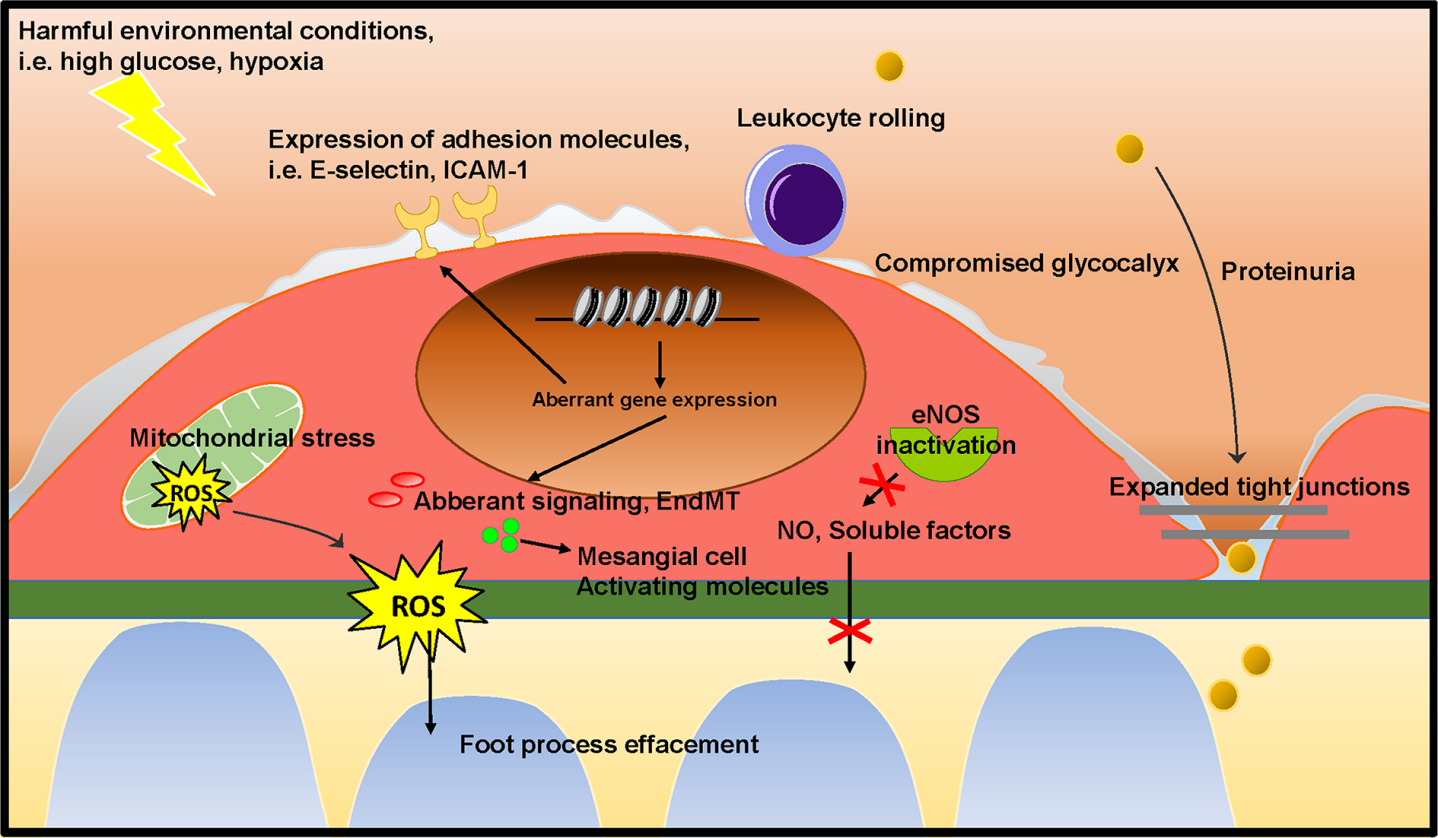
Proposed mechanism on the role of GEnC in the development of glomerular sclerotic diseases. Harmful environmental conditions, such as hyperglycemia and hypoxia cause GEnC dysfunction. GEnC dysfunction is characterized by a compromised endothelial glycocalyx, an inflammatory phenotype, mitochondrial damage and oxidative stress, aberrant signaling and EndMT, resulting in proteinuria, podocyte damage or loss, mesangial activation, and ultimately glomerulosclerosis.

As outlined above, podocytes and mesangial cells have previously received a lot of attention in research on the pathogenesis of FSGS and DN. However, the studies summarized in this review show that GEnC dysfunction occurs in the early stages of FSGS and DN, and contributes to podocyte damage and mesangial activation, eventually culminating in glomerulosclerosis. Several of the studies described here show that GEnC dysfunction precedes podocyte damage, and is sufficient to develop proteinuria. This provides a new insight on the role of GEnCs in the early phase in development of FSGS and DN. GEnC dysfunction is characterized by a compromised endothelial glycocalyx, an inflammatory phenotype, mitochondrial damage and oxidative stress, aberrant signaling and EndMT, resulting in proteinuria, podocyte damage or loss, mesangial activation, and ultimately glomerulosclerosis (**Figure 4**). The glomerular endothelium poses a potential efficacious cellular target to pharmacologically halt disease development and progression in DN and FSGS. Aberrant gene expression patterns largely contribute to GEnC dysfunction and altered epigenetic mechanisms seem involved in this aberrant transcriptome. To expand our understanding of the cross-talk between GEnCs and other glomerular cells in health and disease, isolated systems could be useful, such as co-cultured cells and organoids. Co-culture systems of differentiated GEnCs and podocytes (Li et al., 2016) and organoids (Hale et al., 2018) with subsequent endothelial genetic and epigenetic characterization and manipulation could be instrumental for understanding the pathways involved in GEnC-podocyte cross-talk. Until now, the knowledge of the epigenetic mechanisms involved in GEnC dysfunction in DN and FSGS is scarce and needs to be expanded.

Transcriptome profiling of GEnCs in DN and FSGS is of utmost importance to identify aberrantly expressed genes and associated regulatory pathways. Epigenomic databases, such as encyclopedia of DNA elements (ENCODE), in which chromatin modifications on both DNA and histone proteins are mapped in various cell lines (Consortium, 2012), could reveal potential epigenetic modifications responsible for aberrant expression patterns. Cell-specific delivery is needed to therapeutically intervene in the epigenetic mechanisms involved in GEnC dysfunction to avoid off-target cell effects. The identification of epigenetic mechanisms involved in GEnC dysfunction can effectively be studied with CRISPR-Cas9 technology *in vitro* (Adli, 2018). However, cell-specific delivery of CRISPR-Cas is still a huge challenge (Adli, 2018). The delivery of nucleotides, such as siRNAs therefore is an approach with great potential for intervention in GEnCs. As epigenetic modifications are regulated by epigenetic enzymes, intervening in the expression of epigenetic enzymes can influence the amount of epigenetic modifications. Endothelial cell-specific delivery of siRNA is feasible and this strategy has previously been used to successfully deliver siRNA to inflamed endothelial cells, including specifically GEnCs, and to decrease the expression of the target gene of interest (Kowalski et al., 2014; Choi et al., 2017).

## Author Contributions

MS searched articles, drafted and wrote the manuscript. MS, JK, GK, and J-LH created the outline of the manuscript. JK, JB, MH, JV, GK, and J-LH supervised the manuscript writing and revised the manuscript. All authors contributed to the article and approved the submitted version.

## Funding

This study was financially supported by the Dutch Kidney Foundation (grant 15OP13).

## Conflict of Interest

The authors declare that the research was conducted in the absence of any commercial or financial relationships that could be construed as a potential conflict of interest.
